# Supplementary material for: Estimating growth patterns and driver effects in tumor evolution from individual samples
Source: Nat Commun. 2020 Feb 5;11:732. doi: 10.1038/s41467-020-14407-9 (PMC7002450; doi:10.1038/s41467-020-14407-9)
Supplement: Supplementary file 2 — Supplementary Information [file 41467_2020_14407_MOESM2_ESM.pdf]

Estimating growth patterns and driver effects in tumor evolution from individual samples

Salichos et al. 2019

Supplementary Notes

Supplementary note 1. Variant allele frequency (VAF): The fraction of sequencing reads overlapping a genomic coordinate that support the non-reference allele. This fraction can be further normalized based on the sample's ploidy and purity.

Supplementary note 2. Variant call format (VCF) file: A text file format that includes sequencing information such as the position and frequency of every mutation in the sample.

Supplementary note 3. Subclone: Cells that belong to a single lineage during population growth. Within the subclone, a higher mutational frequency is associated with an earlier time of I.

Supplementary note 4. Linear subclones: Population growth where every subclone has at most one child subclone (e.g., Subclone A -> Subclone B -> Subclone C).

Supplementary note 5. Fitness mutation**:** A mutation that increases the growth of the population. Typically, a fitness mutation might lead to the formation of a subclone. A fitness mutation does not necessarily induce tumorigenesis.

Supplementary note 6. PCAWG drivers[1]**:** In our analysis, we used state-of-the-art driver detection by the PCAWG consortium.

Supplementary note 7. Generational hitchhiker (g-hitchiker): A hitchhike mutation that occurred before the fitness mutation. These mutations have increased VAF (higher than their respective fitness mutation) and represent generational time as their respective branching lineages typically have low VAF (see Figure 1).

Supplementary note 8. Growth *r***:** Before a fitness mutation, the population grows at a rate *r*. In our model, we used the prevalence of generational hitchhikers to estimate growth *r*.

Supplementary note 9. Scalar effect k_i: After the fitness mutation *i* occurs, the population grows at rate $k_i \times r$.

Supplementary note 10. Projected scalar effect k*: Scalar effect k is projected by considering a larger population size when implementing our method directly in equation

$$f_{\mathrm{g}}(T, t_{\mathrm{g}}, t_{\mathrm{i}} - m) = \frac{\mathrm{e}^{-r(t_{\mathrm{g}} + t_{\mathrm{i}} - m)} \times (N_{\mathrm{tot}} - f_{\mathrm{d}(T,t_i)} \times N_{\mathrm{tot}} + \sqrt[k_{\mathrm{i}}]{f_{\mathrm{d}(T,t_{\mathrm{i}})} \times N_{\mathrm{tot}}}) + f_{\mathrm{d}(T,t_{\mathrm{i}})} \times N_{\mathrm{tot}} - \sqrt[k_{\mathrm{i}}]{f_{\mathrm{d}(T,t_{\mathrm{i}})} \times N_{\mathrm{tot}}}}{N_{\mathrm{tot}}} [1].$$

In our script the user is allowed to enter their own estimate of population size N_{tot} to obtain projected k values.

Supplementary note 11. Projected selection coefficient s*: Similar to k*, we use population genetics theory to project simulated selection coefficient s for larger population sizes.

Supplementary note 12. Frequency F_{i}: The frequency of mutation i at the time of sequencing.

Supplementary note 13. Frequency function f_{g} (t_{g}, $t_{\mathrm{i-m}}$, r, k): The function that describes the frequency F_{i-m} for m g-hitchhikers occurring before the fitness mutation i.

Supplementary note 14. Generational time t_{g}: A time specific re-optimized constant to calibrate generational time for m respective g-hitchhikers. This is a very important twist of our method, allowing to localize the effect timewise without considering past events including copy number variations or other VAF perturbations.

Supplementary note 15. Growth vector: For each mutation $i > m$ in the tumor sample, we estimated growth r_{i-1}.

Supplementary note 16. Effect vector: For each mutation $i > m$ in the tumor sample, we estimated its fitness effect k_{i}.

Supplementary note 17. Peak vector: Local peaks for a vector that correspond to fitness mutations with highest growth effect $k_{\mathrm{i}} \times r_{\mathrm{i-1}}$.

Supplementary note 18. Optimizing function: For m g-hitchhikers occurring before mutation i, we used a nonlinear least square fitting to calculate effect k_i and generational time t_{g}.

Supplementary note 19. Vogelstein cancer genes[2]**:** Our Vogelstein list consists of 71 tumor suppressor genes and 54 oncogenes.

Supplementary note 20. Tumor Suppressor Genes from Vogelstein list: *ACVR1B, APC, ARID1A, ARID1B, ARID2, ASXL1, ATM, ATRX, AXIN1, B2M, BAP1, BCOR, BRCA1, BRCA2, CASP8, CDC73, CDH1, CDKN2A, CEBPA, CIC, CREBBP, CYLD, DAXX, EP300, FAM123B, FBXW7, FUBP1, GATA1, GATA3, HNF1A, KDM5C, KDM6A, MAP3K1, MEN1, MLH1, MLL2, MLL3, MSH2, MSH6, NCOR1, NF1, NF2, NOTCH1, NOTCH2, NPM1, PAX5, PBRM1, PHF6, PIK3R1, PRDM1, PTCH1, PTEN, RB1, RNF43, RUNX1, SETD2, SMAD2, SMAD4, SMARCA4, SMARCB1, SOCS1, SOX9, STAG2, STK11, TET2, TNFAIP3, TRAF7, TP53, TSC1, VHL, WT1*

Supplementary note 21. Oncogenes from Vogelstein list: *ABL1, AKT1, ALK, AR, BCL2, BRAF, CARD11, CBL, CRLF2, CSF1R, CTNNB1, DNMT1, DNMT3A, EGFR, RBB2, EZH2, FGFR2, FGFR3, FLT3, FOXL2, GATA2, GNA11, GNAQ, GNAS, H3F3A, HIST1H3B, HRAS, IDH1, IDH2, JAK1, JAK2, JAK3, KIT, KLF4, KRAS, MAP2K1, MED12, MET, MPL, MYD88, NFE2L2, NRAS, PDGFRA, PIK3CA, PPP2R1A, PTPN11, RET, SETBP1, SF3B1, SMO, SPOP, SRSF2, TSHR, U2AF1*

Supplementary note 22. Random gene list, comparable to Vogelstein gene list. To create a 'random gene list' comparable to the Vogelstein gene list, we randomly selected non-Vogelstein genes that had a similar number of mutations in the PCAWG database. *SLCO1B1, PDZD4, OPA1, ABCC9, FRAS1, PSME4, MYCBP2, DCAF4L2, GRID2, OR2G6, NALCN, MYLK, ITGA10, ASAP3, ZNF844, CNTNAP4, WDR90, ADAMTS20, CDH17, TRPM3, FLT1, LY9, GJA8, MAT1A, SLCO1A2, RBP3, GOLGA7, FANCM, DYSF, GNAO1, ADAMTS8, MXRA5, APBA1, RNF214, NHSL1, SYT7, MYC, NBEAL2, DDI1, GPR116, CNTN1, PASD1, PHLPP2, FAM47B, MAGEF1, PLOD1, KDM4E, RXRB, KIAA1211L, HSD3B7, C12orf54, ERBB4,*

ADIPOQ, GFAP, SLC5A7, BAIAP2L1, KIF7, ATHL1, BEST3, PLXNC1, MROH7, KCNH8, SYCP2, CYFIP2, ARHGEF16, FLG, ZFX, ITGA4, CXorf22, BTK, PREX1, PKN2, FILIP1L, CPXCR1, OSBPL6, KCNH1, COL21A1, ABCB5, NACA, PLCL1, ZNF804A, PLCB1, HMSD, ARHGEF4, DSG3, PCDHB4, PCDHA4, ARHGDIB, ANK3, ADAMTS10, THBS2, WNK2, EML6, PIM1, PCSK5, MUC22, MGA, LRRIQ1, FN1, HRNR, MYH13, LPHN2, TNC, PTPRZ1, PKD1L1, ASPM, KCNQ3, CENPF, KCNT2, VPS13C, VNN3, NWD1, AKAP9, KIAA1549, C10orf71, MUC16, SGK1, GRM3, HSPG2, ZFHX3, FREM3, CDH10S

<u>Supplementary note 23. On tumor linearity</u>: To minimize subclonal entanglements that could affect our calculations and to facilitate our sliding window analysis, we selected 993 whole genome sequenced tumors from PCAWG that were linear, in that no subclone had two children subclones based on PhyloSub[3]. PhyloSub provides the clonal branching history, allowing us to determine of clonal evolution. However, our method could also be applied to early (parent) subclones.

Supplementary Methods

<u>Simulation analysis using the Gillespie algorithm</u>. We used a stepwise time-branching process to model the growth of a single transformed cell into a tumor with a dominant subclone. The workhorse of our simulations is the Gillespie algorithm[4], which has frequently been used to simulate stochastically dividing cells. In the simulations of our main analyses, there are two kinds of cells: clonal cells and driver subclone cells, where driver subclone cells carry an additional driver not present in the original tumor cell of a simulation. Each run of a simulation proceeds as a series of events until the stop condition is met. Each event has an associated event type, parental cell, and duration, and each of these three attributes of the event are drawn

randomly. In the simulations of our main analyses, there are 5 possible event types: 1) one clonal cell divides into two clonal cells; 2) one clonal cell divides into a clonal cell and a subclonal cell; 3) one subclonal cell divides into two subclonal cells; 4) a clonal cell dies; and 5) a subclonal cell dies. To determine which event type is associated with a given event, one of the event types is sampled at random, according to weights that reflect the state of the tumor.

The weight for event type 1 (one clonal cell becoming two clonal cells) is the sum of the birth rates of all clonal cells, which is in turn typically 1; hence, the weight for event type 1 is typically equal to the number of clonal cells in the tumor at a given time. Similarly, the weight for event type 3 (one subclonal cell becoming two subclonal cells) is the sum of the birth rates of all subclonal cells, which is in turn typically k; hence, the weight for event type 3 is typically k times the number of subclonal cells in the tumor at a given time. The weight for event type 4 (the death of a clonal cell) in the main analyses follows a logistic paradigm: the total number of cells in the tumor, divided by the tumor's carrying capacity, (which gives the death rate of a single cell) and then multiplied by the number of clonal cells in the tumor (which gives the total death rate across all clonal cells). The weight for event type 5 (the death of a subclonal cell) is identical except that the number of subclonal cells is used in place of the number of clonal cells. Event type 2 (one clonal cell becomes one clonal cell and one subclonal cell) is special and occurs only once per simulation when some threshold minimum number of mutations per cell has been achieved. This ensures good mutation accumulation. Once this threshold is reached, event type 2 has a 10% chance of occurring per turn. Event type 2 has also a weight of 0 once the subclonal driver has appeared. If the last surviving cell of the subclone would be killed by a sampled event type, the event type is re-rolled. Event types 1, 2, and 3 involve the splitting of a cell into two

cells. These two cells inherit all the mutations of the parental cell and, in the main analysis, acquire one new mutation as well.

Each event type is associated with one parental cell type, with some redundancy. Event types 1, 2, and 4 involve a clonal parental cell type. Event types 3 and 5 involve a subclonal parental cell type. The parent cell for the event is randomly drawn from all cells that match the involved parental cell type, with uniform weights assigned to the various instances of that cell type. The duration of the event (or rather, the time elapse between the preceding event and the current event) is sampled from the exponential decay function with a mean equal to the reciprocal of the sum of the weights of all event types, in accordance with the Gillespie algorithm. Effectively, this method samples time frequently when the tumor is large and subject to high rates of birth and death, and samples time infrequently when the tumor is small or slow. The simulation ends after the driver subclone reaches a critical prevalence.

Neutral and non-neutral simulations based on Williams et al. 2016, 2018[5,6]. To benchmark our model on an independent simulation dataset, we applied our method on a) 140 neutral simulations of tumor progression and b) 360 non-neutral simulations for various growth scenarios, generated from the validated simulation software for neutral tumors from Williams et al. 2016 and for non-neutral tumors from Williams et al. 2018. These scripts have the advantage of being existing, validated tools, but the limitation of being constrained by the models used by their authors. In both the neutral and non-neutral tumors, the tumor starts as a single transformed cell, which as with its descendants, divides stochastically to form a growing tumor. Each cell division was set to produce an average of 10 mutations per haploid genome, and read depth of simulated sequencing was 1000x. For the non-neutral tumors, the probability of division a cell in the fitter subclone is modified by a selection coefficient drawn from a complex distribution

determined by the package. Subclones were grown to either low, medium, or high prevalence corresponding to prevalence ranges 0.1 to 0.2, 0.2 to 0.3, and 0.3 to 0.4 VAFs, respectively. For non-neutral growth we used CancerSeqSim, while for neutral growth we used 'neutral-tumor-evolution' packages. For neutral and non-neutral growth, we used mutations with min true VAF 0.01 and 0.05 respectively. In these analyses, simulated drivers correspond to a pre-chosen $1+s$ selection coefficient, while scalar k represents our method's predictions. We also used population projections to increased cell-population sizes up to 1 billion cells. Coefficients s^* and scalars k^* represent projected values to higher populations sizes. For calculating s^* we used population genetic models (see below), while for k^* we modified the population size in our method's code. However, when running our code, the user can provide their own population size estimate, either using the number of mutations as proxy, or by an intelligent guess. Varying population sizes did not burden our method's detectability, but do provide a decreased s^* and k^* as expected. Our default analysis included a population size of 10,000 cells, medium VAFs, a range of selection coefficients between $0 < s < 34$, a sequencing coverage of 1000x and an optimal hitchhiker sliding window size of 150 mutations. The hitchhiker window size was optimized using our neutral simulations and a range of window sizes until their median effect peaks has a median of 1, for the corresponding population size and sequencing coverage. A sliding window size of 100 hitchhiker mutations provided higher predicted scalar effects k for both neutral and non-neutral simulations, without burdening our method's ability to detect drivers.

Supplementary Figures

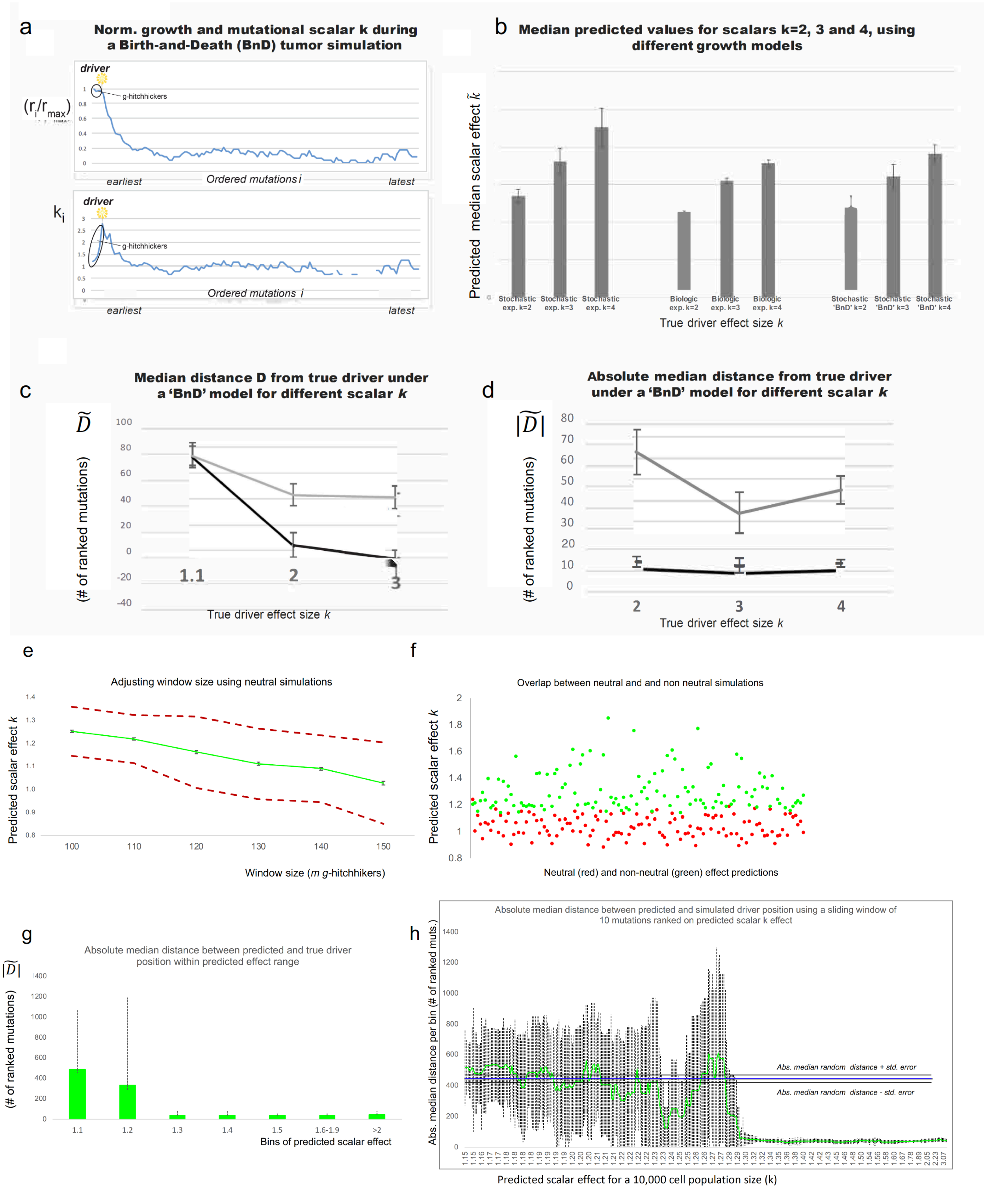
a
Norm. growth and mutational scalar k during a Birth-and-Death (BnD) tumor simulation
driver
g-hitchhickers
(r_i/r_{max})
earliest
Ordered mutations i
latest
k_i
b
Median predicted values for scalars k=2, 3 and 4, using different growth models
Predicted median scalar effect $\tilde{k}$
Stochastic exp. k=2
Stochastic exp. k=3
Stochastic exp. k=4
Biologic exp. k=2
Biologic exp. k=3
Biologic exp. k=4
Stochastic 'BnD' k=2
Stochastic 'BnD' k=3
Stochastic 'BnD' k=4
True driver effect size k
c
Median distance D from true driver under a 'BnD' model for different scalar k
$\widetilde{D}$
(# of ranked mutations)
True driver effect size k
d
Absolute median distance from true driver under a 'BnD' model for different scalar k
$|\widetilde{D}|$
(# of ranked mutations)
True driver effect size k
e
Adjusting window size using neutral simulations
Predicted scalar effect k
Window size (m g-hitchhikers)
f
Overlap between neutral and and non neutral simulations
Predicted scalar effect k
Neutral (red) and non-neutral (green) effect predictions
g
Absolute median distance between predicted and true driver position within predicted effect range
$|\widetilde{D}|$
(# of ranked mutations)
Bins of predicted scalar effect
h
Absolute median distance between predicted and simulated driver position using a sliding window of 10 mutations ranked on predicted scalar k effect
Abs. median distance per bin (# of ranked muts.)
Abs. median random distance + std. error
Abs. median random distance - std. error
Predicted scalar effect for a 10,000 cell population size (k)

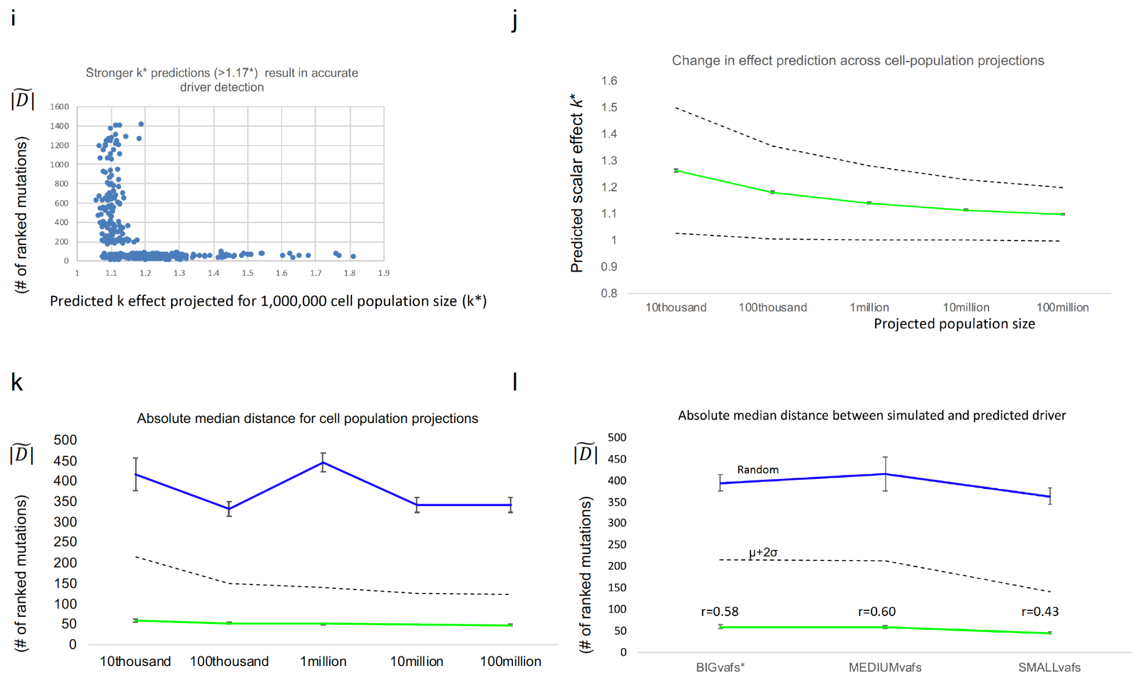

Supplementary figure 1. Predicting driver position and scalar effect k using simulations. To test our model, we used various types of simulations with sampling noise including exponential stochastic growth, exponential growth with biological restrictions in replication timing and birth and death (BnD) Gillespie stochastic model. For every simulation, we introduced a driver mutation with a true/known fitness effect. Further, we "sequenced" the population assigning each mutation with a population frequency. Then, based on mutational frequencies we estimated the predicted driver's position and effect (across the ordered mutations). By 'distance from true driver' we denote the number of mutations between the true and the predicted driver position. In **a)** we show the predicted growth and effect peak (simulated effect *k*=3) for one simulation, where mutations are ordered based on their frequency and the predicted peak corresponds to the exact position of the true driver. In **b)** We show the median predicted value across a range of k effect sizes using three different growth models**. c)** To test if we can significantly predict the driver's position/timing, we measured the median and **d)** the absolute median distance (as in number of mutations) between predicted and true driver**.** We were able to successfully approximate the driver's position (in black) compared to random (in grey) for various effect sizes. In **e)** Using Julie software from Williams et al 2016, we generated 140 neutral simulations of tumor progression for a population of 10000

cells. Neutral peaks using an optimal window size of m=150 hitchhikers had a median scalar effect k of 1.03, $2\times\sigma$=0.18 (dotted lines) and median standard error equal to 0.01 (capped bars). In **f)** we show the overlap between neutral and non-neutral simulations. Scalar effect predictions for neutral (red dots) and non-neutral (green dots) simulations showed a small overlap. Neutral effect peaks have a median $\tilde{k}$ equal to 1.03. In **g)** we show that stronger drivers result in accurate detection of driver's position (within effect range)**.** By implementing the Williams et al 2018 algorithm for stochastic tumor progression we simulated 360 tumor progressions with a populations size of 10000 cells**.** We estimated the absolute median distance (and 95% deviation) between the simulated and predicted driver using bins of various scalar k effect sizes. Dotted lines represent a 2×sigma deviation (95%). When our method predicted a higher than 1.29 scalar k for the specific population size, driver detection became highly accurate. For random mutations selected from the same samples the absolute median distance is 444.5, with a standard error of the median ±24.5. In **h)** after ranking simulations based on the predicted scalar effect k for every simulation (from smallest to highest effect) we used a sliding window of size 20 to estimate the absolute median distance (and 95% deviation) between the simulated and predicted driver per bin of 20. Dotted lines represent a 2×sigma deviation (95%). When our predicted scalar effect k was higher than 1.29 our driver detection was highly accurate. Blue line represents our random absolute median distance (444.5), while black lines represent the standard error of the median for these expectation (±24.5). In **i**) Each dot represents a single simulation. We plot the absolute distance between simulated and predicted driver in association with the predicted effect k^*. A large effect denotes the presence of a driver with great accuracy (small $|D|$). In **j)** we show the predicted driver effect across various population projections. By implementing the Williams et al 2018 algorithm for stochastic tumor progression we simulated 360 tumor progressions with a populations size of 10000 cells. By directly modifying the total population size in equation [1] in our algorithm, we then predicted the drivers' median effect by projecting onto larger population sizes. Capped error bars represent the standard error of the median, while dotted lines represent a $2\times\sigma$ deviation (95%). Adjusting our model for larger population sizes decreased the scalar

effect prediction**.** In **k)** similarly to (j) we also predicted the driver position in larger population sizes. Green line represents the absolute median distance (as in number of ranked mutations) between predicted and simulated drivers. Blue line represents the absolute median distance between each simulation's random prediction and the simulated driver. Capped error bars represent the median standard error, while dotted lines represent a $2\times\sigma$ deviation (95%). Adjusting our model for larger population sizes did not burden our method. In contrast, our result showed a slight improvement in driver detections. Finally, in **l)** we predicted the driver position for simulated drivers with high, medium or low VAF. Green line represents the absolute median distance between predicted and simulated drivers. Blue line represents the absolute median distance between each simulation's random prediction and the simulated driver. Simulated drivers with smaller allele frequencies showed a lower potential in predicting the driver's effect (lower correlation between simulated and predicted effect). Interestingly, they also provided driver detections with higher accuracy (absolute median distance between simulated and predicted driver equal to 46 ranked mutations, compared to 60 and 59.5 for higher and medium VAFs).

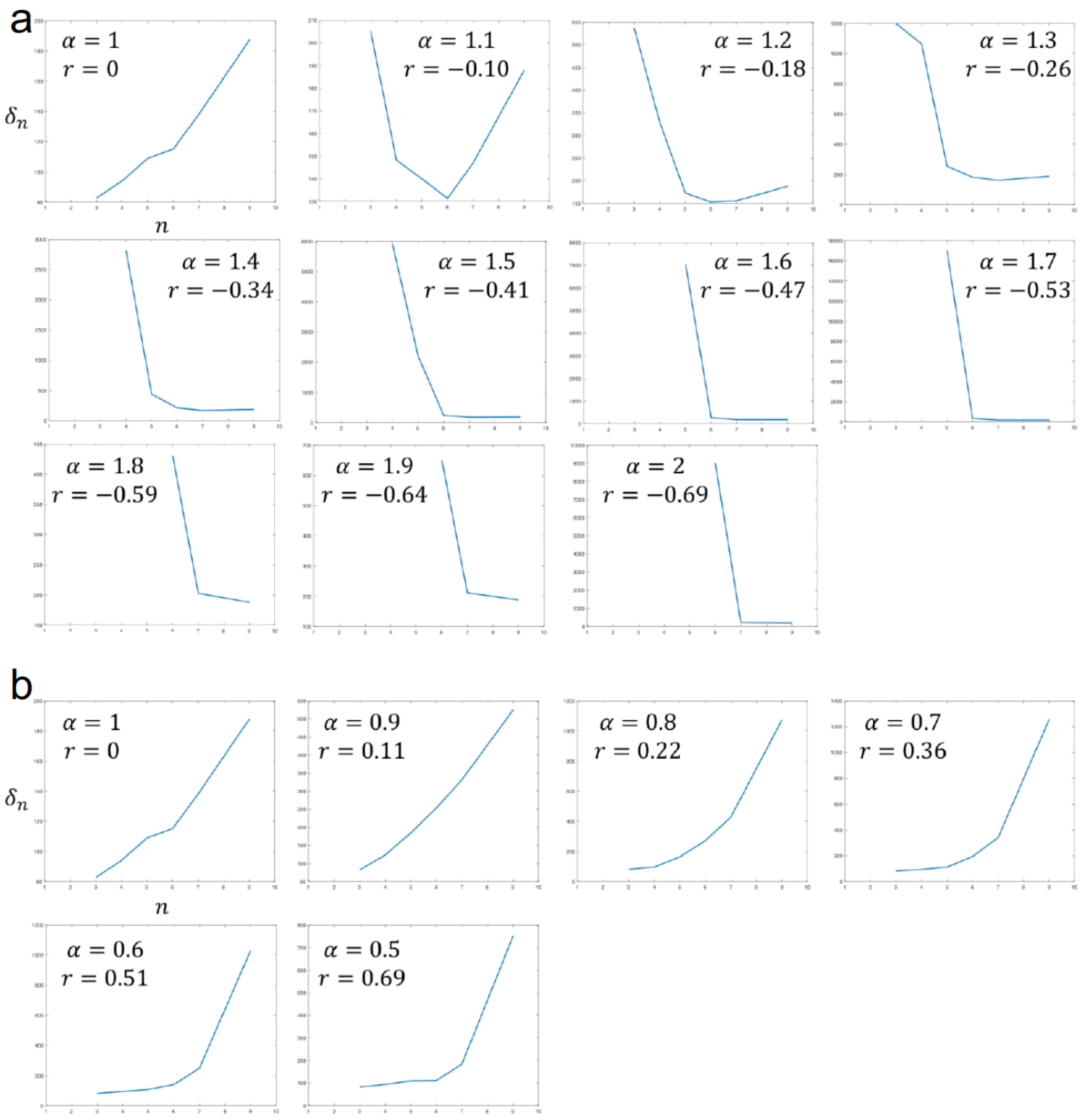

Supplementary figure 2. Using Kingman's coalescent theory, for a length of time T_n with n lineages, we show that the growth $\hat{r}$ estimator remains qualitatively unchanged (positive or negative) even for non g-hitchhikers. By approximation, the mutational density δ_n within windows [1/n 1/(n − 1)), whose lengths are L_n is equal to $\delta_n = \frac{M_n}{L_n} \propto 2\mu n$. As mutational density δ_n increases with n, and hence with time, $\hat{r}$ estimator is predicted to take positive values for both constant and varying size populations. Similarly, for negative growth values, density δ_n decreases with time. A small positive bias is observed in

cases of growth r=0, as the pattern reverses. Using a population model $N^{t+1} = \alpha N^{t}$, we let **(A)** $\alpha > 1$ corresponding to a decreasing population (time is indexed in reverse) and **(B)** $\alpha < 1$ corresponding to an increasing population.

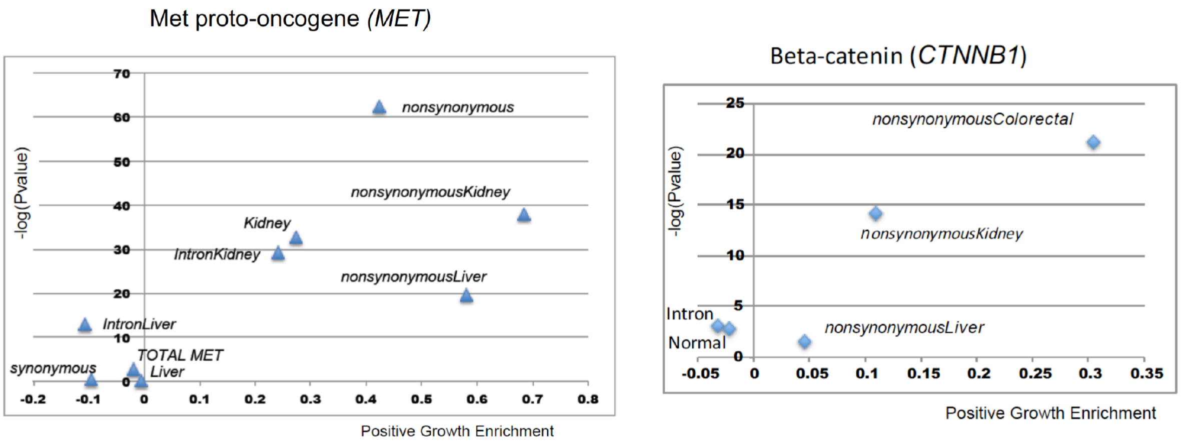

Supplementary figure 3. Both *MET* and *CTNNB1* genomic regions appear to be slightly depleted during periods of positive growth, whereas nonsynonymous mutations show positive associations for specific cancers. The x-axis represents growth enrichment, while the y-axis shows the level of significance as the negative logarithm of a two tailed t-test P value (-log(p-value).

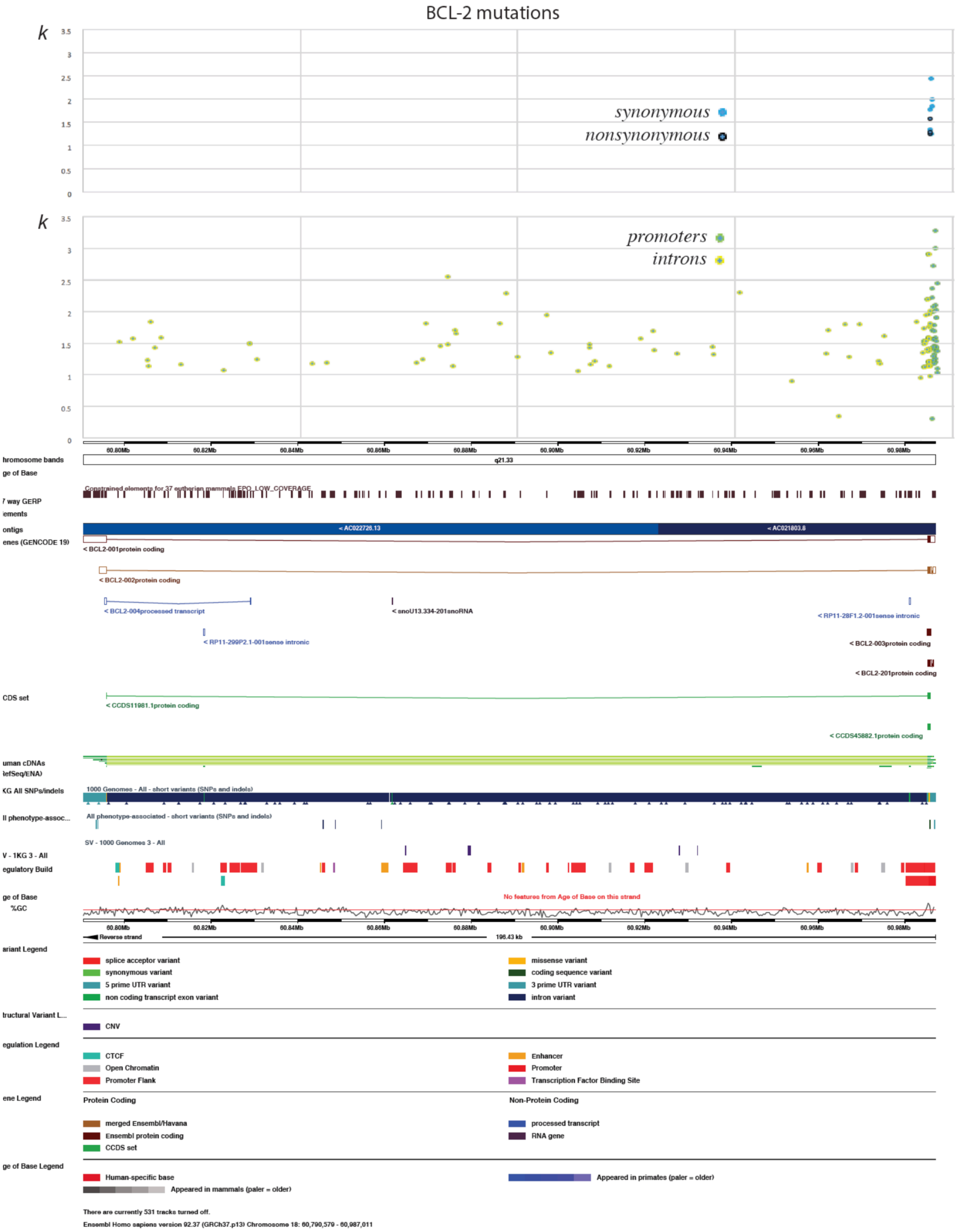

Supplementary figure 4. Mapping of missense, nonsynonymous, promoter, synonymous and intronic mutations from 993 tumor samples across the *BCL2*'s genomic region. Interestingly, synonymous

mutations placed at an early mutational hotspot was associated with periods of positive growth. Genomic profile for *BCL2* was obtained from ENSEMBL (http://www.ensembl.org).

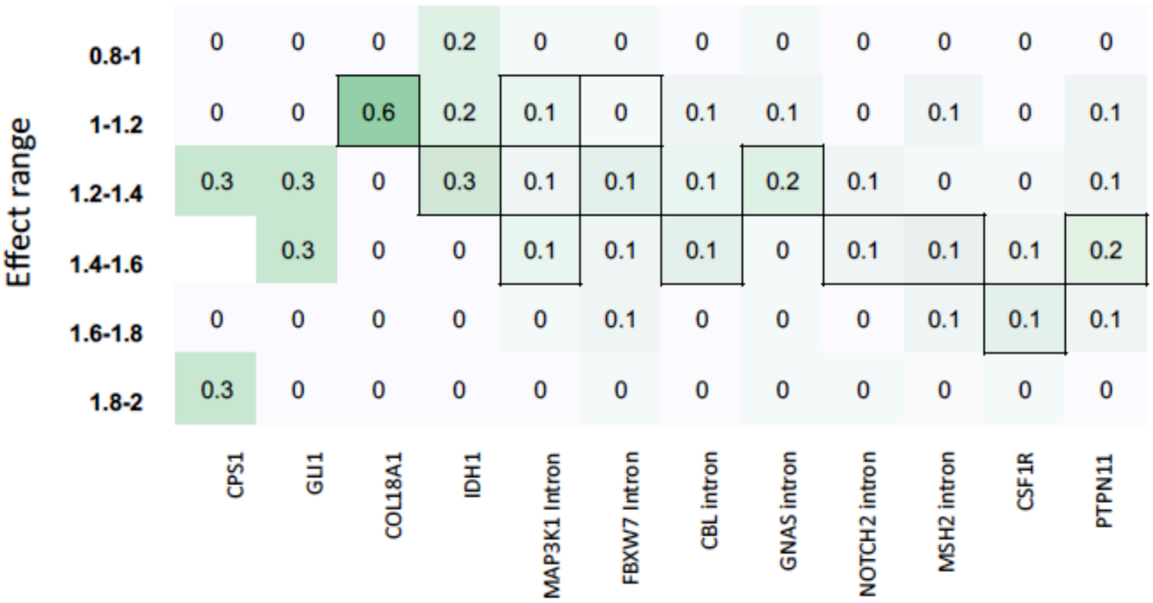

Supplementary figure 5. Using 993 tumor samples, we identified candidate genes that were associated with positive growth from an AML ultra-deep sequenced tumor that showed an overall positive association with positive growth for enrichment different effect bins. Dark boxes denote significance for the specific effect range/bin using a two tailed t-test and P<0.00001.

Supplementary References

1. Sabarinathan, R. *et al.* The whole-genome panorama of cancer drivers. *bioRxiv* (2017). doi:10.1101/190330
2. Vogelstein, B. *et al.* Cancer genome landscapes. *Science* **339**, 1546–58 (2013).
3. Jiao, W., Vembu, S., Deshwar, A. G., Stein, L. & Morris, Q. Inferring clonal evolution of tumors from single nucleotide somatic mutations. *BMC Bioinformatics* (2014). doi:10.1186/1471-2105-15-35
4. Gillespie, D. T. A general method for numerically simulating the stochastic time evolution of coupled chemical reactions. *J. Comput. Phys.* (1976). doi:10.1016/0021-9991(76)90041-3
5. Williams, M. J. *et al.* Quantification of subclonal selection in cancer from bulk sequencing

data. *Nat. Genet.* (2018). doi:10.1038/s41588-018-0128-6
6. Williams, M. J., Werner, B., Barnes, C. P., Graham, T. A. & Sottoriva, A. Identification of neutral tumor evolution across cancer types. *Nat. Genet.* (2016). doi:10.1038/ng.3489
