# Supplementary material for: Estimating growth patterns and driver effects in tumor evolution from individual samples
Source: Nat Commun. 2020 Feb 5;11:732. doi: 10.1038/s41467-020-14407-9 (PMC7002450; doi:10.1038/s41467-020-14407-9)
Supplement: Supplementary file 3 — Description of Additional Supplementary Files [file 41467_2020_14407_MOESM3_ESM.pdf]

Descriptions of Additional Supplementary Files

File Name: Supplementary Data 1
Description: Positive Enrichment and δk for 293 genes.

File Name: Supplementary Data 2
Description: Gene ontology (GO) enrichment for genes with positive growth during tumor progression.
