# Supplementary material for: Estimating growth patterns and driver effects in tumor evolution from individual samples
Source: Nat Commun. 2020 Feb 5;11:732. doi: 10.1038/s41467-020-14407-9 (PMC7002450; doi:10.1038/s41467-020-14407-9)
Supplement: Supplementary file 6 — Reporting Summary [file 41467_2020_14407_MOESM6_ESM.pdf]

natureresearch

Corresponding author(s): Mark Gerstein

Last updated by author(s): Oct 11, 2019

Reporting Summary

Nature Research wishes to improve the reproducibility of the work that we publish. This form provides structure for consistency and transparency in reporting. For further information on Nature Research policies, see Authors & Referees and the Editorial Policy Checklist.

Statistics

For all statistical analyses, confirm that the following items are present in the figure legend, table legend, main text, or Methods section.

n/a	Confirmed	
☐	☒	The exact sample size (*n*) for each experimental group/condition, given as a discrete number and unit of measurement
☒	☐	A statement on whether measurements were taken from distinct samples or whether the same sample was measured repeatedly
☐	☒	The statistical test(s) used AND whether they are one- or two-sided<br>*Only common tests should be described solely by name; describe more complex techniques in the Methods section.*
☒	☐	A description of all covariates tested
☐	☒	A description of any assumptions or corrections, such as tests of normality and adjustment for multiple comparisons
☐	☒	A full description of the statistical parameters including central tendency (e.g. means) or other basic estimates (e.g. regression coefficient) AND variation (e.g. standard deviation) or associated estimates of uncertainty (e.g. confidence intervals)
☐	☒	For null hypothesis testing, the test statistic (e.g. *F*, *t*, *r*) with confidence intervals, effect sizes, degrees of freedom and *P* value noted<br>*Give P values as exact values whenever suitable.*
☒	☐	For Bayesian analysis, information on the choice of priors and Markov chain Monte Carlo settings
☒	☐	For hierarchical and complex designs, identification of the appropriate level for tests and full reporting of outcomes
☐	☒	Estimates of effect sizes (e.g. Cohen's *d*, Pearson's *r*), indicating how they were calculated

Our web collection on statistics for biologists contains articles on many of the points above.

Software and code

Policy information about availability of computer code

Data collection	993 Individual VCF files were obtained through PCAWG consortium. PCAWG datasets are available upon request and authorization from the ICGC Data Access Compliance Office and dbGaP10 Authorized Access program for US-based projects, after July 25th 2019. Mutational frequencies for the ultra deep sequenced AML tumor were publicly available. Simulations were generated using custom R scripts, CancerSeqSim and neutral-tumor-evolution packages developed by Williams MJ in 2016 and 2018.
Data analysis	In this study we developed a a framework to identify periods of positive or negative tumor progression and suggest the presence of putative cancer drivers using mutational frequencies from a single VCF file. A perl script that implements this method is provided at https://github.com/gersteinlab/Evotum101. Downstream analysis was performed using custom perl and 'R' scripts.

For manuscripts utilizing custom algorithms or software that are central to the research but not yet described in published literature, software must be made available to editors/reviewers. We strongly encourage code deposition in a community repository (e.g. GitHub). See the Nature Research guidelines for submitting code & software for further information.

Data

Policy information about availability of data

All manuscripts must include a data availability statement. This statement should provide the following information, where applicable:

- Accession codes, unique identifiers, or web links for publicly available datasets
- A list of figures that have associated raw data
- A description of any restrictions on data availability

PCAWG state-of-the-art protected datasets are controlled access that is subject to data usage agreement. PCAWG datasets are available upon request and authorization from the ICGC Data Access Compliance Office and dbGaP10 Authorized Access program for US-based projects, after July 25th 2019. For data repositories and data request see https://docs.icgc.org/pcawg/data/ .
Pseudo-VCF files are provided at https://doi.org/10.6084/m9.figshare.9722651.v1. These files contain real VAF distributions, mutation type information including gene names, but all genomic coordinates and genetic variance have been masked and randomly modified. Data files are provided for figures 2,3,4,5 and

Supplementary figures.

Field-specific reporting

Please select the one below that is the best fit for your research. If you are not sure, read the appropriate sections before making your selection.

☐ Life sciences ☐ Behavioural & social sciences ☒ Ecological, evolutionary & environmental sciences

For a reference copy of the document with all sections, see nature.com/documents/nr-reporting-summary-flat.pdf

Ecological, evolutionary & environmental sciences study design

All studies must disclose on these points even when the disclosure is negative.

Study description	Evolving tumors accumulate thousands of mutations. We have developed a method that quantifies tumor growth and driver effects for individual samples based solely on the VAF spectrum. Drivers introduce a perturbation into this spectrum, and our method uses the frequency of "hitchhiking" mutations preceding a driver to measure this perturbation. Specifically, our method applies various growth models to identify periods of positive/negative growth, the genomic regions associated with them, and the presence and effect of putative drivers. To validate our method, we first used simulation models. Then, we tested our method on 993 linear tumors (i.e. those with linear subclonal expansion, where each parent-subclone has one child) from the PCAWG Consortium and found that the identified periods of positive growth are associated with drivers previously highlighted via recurrence by the PCAWG consortium. Finally, we applied our method to an ultra-deep sequenced AML tumor and identified known cancer genes and additional driver candidates.
Research sample	In our analyses we obtained and used VCF files from 993 individual tumor samples tfrom the PCAWG consortium and one ultra-deep sequenced AML tumor that was already publicly available. PCAWG state-of-the-art protected datasets are controlled access that is subject to data usage agreement. PCAWG datasets are available upon request and authorization from the ICGC Data Access Compliance Office and dbGaP10 Authorized Access program for US-based projects, after July 25th 2019. For data repositories and data request see https://docs.icgc.org/pcawg/data/ .
Sampling strategy	No sample size calculation was performed. The novelty of our method is that driver identification can be suggested on a single individual tumor consisting of thousands of mutations. However, in our work we also used 994 individual tumor samples to derive evolutionary insights about tumor progression. We reported our findings when significant.
Data collection	We obtained access to our dataset through our participation in the PCAWG consortium. PCAWG state-of-the-art protected datasets are controlled access that is subject to data usage agreement. PCAWG datasets are available upon request and authorization from the ICGC Data Access Compliance Office and dbGaP10 Authorized Access program for US-based projects, after July 25th 2019. For data repositories and data request see https://docs.icgc.org/pcawg/data/
Timing and spatial scale	N/a
Data exclusions	N/a
Reproducibility	To benchmark our method we used four different sets of simulations and two sets of real data (a total of 994 tumor samples). Our results are reproducible.
Randomization	This is not applicable to our study. Our method identifies periods of positive growth during tumor progression and suggests the presence of a putative driver. Each individual sample or simulation is an independent evolutionary process.
Blinding	Our study is a theoretical framework that we developed and implemented in-silico on real and simulated data. Blinding does not apply.

Did the study involve field work? ☐ Yes ☒ No

Reporting for specific materials, systems and methods

We require information from authors about some types of materials, experimental systems and methods used in many studies. Here, indicate whether each material, system or method listed is relevant to your study. If you are not sure if a list item applies to your research, read the appropriate section before selecting a response.

Materials & experimental systems

n/a	Involved in the study
☒	☐ Antibodies
☒	☐ Eukaryotic cell lines
☒	☐ Palaeontology
☒	☐ Animals and other organisms
☒	☐ Human research participants
☒	☐ Clinical data

Methods

n/a	Involved in the study
☒	☐ ChIP-seq
☒	☐ Flow cytometry
☒	☐ MRI-based neuroimaging
